# Experimentally constrained CA1 fast-firing parvalbumin-positive interneuron network models exhibit sharp transitions into coherent high frequency rhythms

**DOI:** 10.3389/fncom.2013.00144

**Published:** 2013-10-22

**Authors:** Katie A. Ferguson, Carey Y. L. Huh, Bénédicte Amilhon, Sylvain Williams, Frances K. Skinner

**Affiliations:** ^1^Division of Fundamental Neurobiology, Toronto Western Research Institute, University Health NetworkToronto, ON, Canada; ^2^Department of Physiology, University of TorontoToronto, ON, Canada; ^3^Department of Psychiatry, Douglas Mental Health University Institute, McGill UniversityMontreal, QC, Canada; ^4^Departments of Medicine (Neurology) and Physiology, University of TorontoToronto, ON, Canada

**Keywords:** mathematical model, inhibitory networks, basket cells, hippocampus, fast gamma

## Abstract

The coupling of high frequency oscillations (HFOs; >100 Hz) and theta oscillations (3–12 Hz) in the CA1 region of rats increases during REM sleep, indicating that it may play a role in memory processing. However, it is unclear whether the CA1 region itself is capable of providing major contributions to the generation of HFOs, or if they are strictly driven through input projections. Parvalbumin-positive (PV+) interneurons may play an essential role in these oscillations due to their extensive connections with neighboring pyramidal cells, and their characteristic fast-spiking. Thus, we created mathematical network models to investigate the conditions under which networks of CA1 fast-spiking PV+ interneurons are capable of producing high frequency population rhythms. We used whole-cell patch clamp recordings of fast-spiking, PV+ cells in the CA1 region of an intact hippocampal preparation *in vitro* to derive cellular properties, from which we constrained an Izhikevich-type model. Novel, biologically constrained network models were constructed with these individual cell models, and we investigated networks across a range of experimentally determined excitatory inputs and inhibitory synaptic strengths. For each network, we determined network frequency and coherence. Network simulations produce coherent firing at high frequencies (>90 Hz) for parameter ranges in which PV-PV inhibitory synaptic conductances are necessarily small and external excitatory inputs are relatively large. Interestingly, our networks produce sharp transitions between random and coherent firing, and this sharpness is lost when connectivity is increased beyond biological estimates. Our work suggests that CA1 networks may be designed with mechanisms for quickly gating in and out of high frequency coherent population rhythms, which may be essential in the generation of nested theta/high frequency rhythms.

## Introduction

High frequency oscillations (HFOs; >100 Hz) are recorded from the CA1 region of the hippocampus, and are distinct from gamma oscillations (30–100 Hz), sharp-wave ripple oscillations (100–250 Hz), and the spectral leakage of spiking activity (Scheffer-Teixeira et al., 2012). These high frequency rhythms are nested within the slower theta oscillations (3–12 Hz) in the CA1 region during decision making and REM sleep of rats (Tort et al., 2008; Scheffer-Teixeira et al., 2012), and therefore may play an important role in memory processing. However, whether these oscillations are generated by an intrinsic CA1 mechanism or are driven by CA3 and entorhinal cortical projections, and whether the oscillations are generated by a particular cell type or a network of various cell populations, remains unclear.

Insight to these questions have been gained by Jackson et al. (2011), who recorded rhythms in the subiculum with frequencies similar to HFOs. These oscillations were dependent on fast γ-Aminobutyric acid (GABA) inhibition, and not α-Amino-3-hydroxy-5-methyl-4-isoxazolepropionic acid (AMPA), kainite, or N-Methyl-D-aspartic acid (NMDA) glutamate receptors. Thus, a network of inhibitory interneurons may be responsible for the generation of these HFOs. The fast-firing properties of hippocampal parvalbumin-positive (PV+) interneurons, and their extensive, often perisomatic and axo-axonic targeting, connections with neighboring excitatory neurons, provide them with enormous potential to influence hippocampal network rhythms. Indeed, perisomatic targeting interneurons are thought to critically influence the timing of pyramidal cell spiking (Cobb et al., 1995; Miles et al., 1996) and the synchronization of large groups of pyramidal cells (Freund and Buzsáki, 1996), providing them with the means to influence the frequency and power of network oscillations. Thus, fast spiking interneurons likely play an important role in these HFOs.

Mathematical models may provide insight to the underlying mechanisms of these oscillations, as they allow one to parse out specific cell populations and investigate their network properties in a simplified setting. However, direct links between fast-spiking interneuron models and empirically determined cellular intrinsic and network characteristics do not exist, and this contributes to the challenge of interpreting model insights in a biological setting. In particular, CA1 PV+ fast-spiking models have not been developed, and it is well-known that intrinsic cellular properties can critically control network output (e.g., see Wang, 2010 review, p.1214).

In this paper, we develop and use mathematical models of fast-spiking PV+ interneuron networks to gain insight into whether they are capable of directly contributing to HFOs in the hippocampus. We hypothesize that the generation of population high frequencies in the CA1 sensitively depends on external and internal synaptic input levels to these fast-spiking cells. In order to investigate how this balance of inputs affect a network of fast-spiking PV+ interneurons, we develop and use network models that are tied to experimental work on multiple levels. We construct our individual PV+ cell model based on a modification of the simple Izhikevich model (Izhikevich, 2003), with model parameters directly constrained from *in vitro* recordings of PV+ fast-spiking interneurons in the CA1 region of an intact hippocampal preparation in mice. This same experimental setup produces emergent theta rhythms (Goutagny et al., 2009), and PV+ interneuron recordings during the ongoing rhythm allow us to estimate synaptic currents with which to constrain our model on the network level. Using such constraints together with realistic network size and connectivity estimates, we explore with simulations whether our model networks can give rise to coherent population activity at high frequencies. We find that coherent high frequency population rhythms can be produced, but only for particular balances of inhibitory synaptic conductance strengths and external synaptic drives that include physiologically estimated values in specific ways. In addition, our experimentally constrained networks exhibited sharp transitions from random to coherent firing. This provided the network with the capability of gating in and out of a coherent state, which may be an important aspect for the generation of nested theta/high frequency rhythms. Our work thus predicts that while CA1 fast-firing PV+ interneuron networks have the potential to produce high frequency population rhythms on their own, they may easily be perturbed out of this state.

## Materials and methods

### Experiment

#### Animals

Animals of both sexes (P20-28) were used. In order to visualize PV+ interneurons, transgenic mice were used where a fluorescent protein tdTomato was expressed under the control of the PV promoter (PV-tdTomato mice). In order to generate PV-tdTomato mice, PV-Cre homozygote mice [B6;129P2-Pvalbtm1(cre)Arbr/J, stock number: 008069, the Jackson Laboratory] were mated with Ai9 homozygote mice allowing tdTomato expression in Cre-positive neurons [B6;129S6-Gt(ROSA)26Sortm9(CAG-tdTomato)Hze/J, stock number: 007905, the Jackson Laboratory]. Using immunohistochemistry we confirmed that in PV-tdTomato mice, the majority of tdTomato+ neurons in CA1 stratum oriens express PV (87.6 ± 5.3%; mean ± S.E.M. in 4 animals), indicating a high degree of specificity in these mice. All animals were treated according to protocols and guidelines approved by McGill University and the Canadian Council of Animal Care.

#### Intact hippocampal preparation

The acute preparation containing the intact hippocampus was dissected as described previously (Goutagny et al., 2009). Briefly, after decapitation, the brain was quickly removed from the skull and placed in ice-cold high-sucrose solution, containing (in mM) 252 sucrose, 24 NaHCO_3_, 10 glucose, 3 KCl, 2 MgCl_2_, 1.25 NaH_2_PO_4_, and 1 CaCl_2_ (pH 7.3, oxygenated with 95% O_2_ –5% CO_2_). From a hemisected brain, the septum and hippocampus along with the interconnecting fibers were carefully and rapidly dissected out using microspatulas. The preparation was trimmed with fine scissors to remove any remaining cortical tissue and the septum was cut off. The intact hippocampal preparation was left to rest with the CA1 side facing up in oxygenated room-temperature high-sucrose solution (1 mM CaCl_2_) for 30 min–1 h before recording. The intact preparation from only one hemisphere was used for each animal, and the preparation from either the left or the right hemisphere was chosen randomly for each experiment.

#### Electrophysiological recordings and tdtomato labeling visualization

All electrophysiological recordings were performed at 30 ± 2°C, using artificial cerebrospinal fluid (aCSF) containing (in mM) 126 NaCl, 24 NaHCO_3_, 10 glucose, 4.5 KCl, 2 MgSO_4_, 1.25 NaH_2_PO_4_, 0.4 ascorbic acid, and 2 CaCl_2_ (pH 7.3, oxygenated with 95% O_2_ –5% CO_2_). The intact hippocampal preparation was placed and stabilized in the recording chamber using lead weights. PV+ interneurons located in CA1 stratum oriens/alveus within the middle hippocampus were recorded using the visually guided whole-cell patch-clamp technique. Prior to recording, neurons were identified by tdTomato labeling in the soma by illuminating with a 554-nm wavelength light using a fluorescence system (PTI, Monmouth Junction, NJ). The electrophysiology setup was equipped with an upright BX51W1 Olympus microscope, a 20x water-immersion objective, Nomarsky optics, an infrared camera (Cohu, San Diego, CA), a monochrome digital camera for fluorescence imaging (DAGE-MTI, Michigan City, IN) and a temperature controller (model TC-324B, Warner Instruments, Hamden, CT). Patch pipettes (2.5–4 MΩ) were pulled from borosilicate glass capillaries (Warner Instrument, Hamden, CT) and filled with intrapipette solution containing (mM) 144 K-gluconate, 10 HEPES, 3 MgCl_2_, 2 Na_2_ATP, 0.3 GTP, 0.2 EGTA, adjusted to pH 7.2 with KOH. An Axopatch-1C amplifier (Axon Instruments, Foster City, CA), a microelectrode AC amplifier (A-M Systems, Sequim, WA), a Humbug 60 Hz noise eliminator (Quest Scientific, Vancouver, Canada), an audio monitor (A-M Systems) and pClamp9 software (Molecular Devices, Sunnyvale, CA) were used for recording. All drugs were obtained from Sigma-Aldrich (St. Louis, MO) unless otherwise noted.

For examining intrinsic properties, the oxygenated aCSF was perfused at a relatively fast rate of 20–25 ml/min to ensure the health of the preparation and synaptic blockers were used to inhibit synaptic events [5 μ M 6,7-Dinitroquinoxaline-2,3-dione disodium salt (DNQX), 5 μ M bicuculline and 25 μ M DL-2-Amino-5-phosphonopentanoic acid sodium salt (DL-AP5); Abcam, Toronto, Canada]. Recordings were kept for analysis only if spikes overshot 0 mV (before junction potential correction) and access resistance was < 30 MΩ. Intrinsic properties of each cell were measured in current-clamp mode following published protocols (Huh et al., 2010). The cell's resting membrane potential was measured once the whole-cell configuration was achieved. While the membrane potential of the cell was held at −60 mV in current clamp, a series of small-amplitude 1-s hyperpolarizing steps (10-pA increments) were used to determine the membrane resistance and membrane time constant. A series of 1-s depolarizing current steps (10- and 50-pA increments) from the holding potential of −60 mV were applied, for frequency-current (f-I) analysis.

For simultaneous local field potential (LFP) and whole-cell recording, the oxygenated aCSF was perfused without synaptic blockers at a rate of 20–25 ml/min, which has been tested to be ideal for generation of network theta oscillations in the intact hippocampal preparation (Goutagny et al., 2009). For LFP recordings, a borosilicate-glass field electrode (1–5 MΩ) was placed in CA1 stratum radiatum of middle hippocampus. Once a stable network theta rhythm was detected, whole-cell recordings were performed on PV+ interneurons located in CA1 stratum oriens. For whole-cell recordings, pipette resistance of 2.5–4 MΩ was used. The junction potential was estimated at −15.2 mV, and membrane potentials were corrected for this. Once a stable whole-cell mode was achieved, access resistance and the neuron's resting membrane potential were noted. Then, the cell was recorded at this resting potential together with the LFP signal (containing network theta oscillations) for 60 s, to observe the neuron's spontaneous firing behavior. Next, the neuron's basic properties were quickly checked for, including firing rate, action potential properties, and sag amplitude. Access resistance and resting membrane potential were checked every 5–10 min throughout the recording of the cell. Recordings were kept for analysis only if the LFP signal contained oscillations with frequencies exceeding 2.5 Hz and, as for the intrinsic properties, if the neuron's spikes overshot 0 mV and the access resistance was <30 MΩ.

The reversal potential for inhibitory postsynaptic currents (IPSCs) was determined for CA1 PV+ interneurons using electrical stimulation. For these experiments, aCSF perfusion rate of 7–8 ml/min was used. A monopolar tungsten microelectrode (WPI, Sarasota, FL) was placed on the surface of CA1 (stratum oriens/alveus) in the middle hippocampus. The stimulation parameters were controlled using an isolated pulse stimulator (model A360, WPI). One pulse (25–300 μ A intensity, 0.1 ms duration) was administered every 10 s. CA1 PV+ interneurons located in the middle hippocampus and close to the stimulating electrode were recorded in whole-cell mode. Neurons were held at various potentials in voltage clamp (−100 to +30 mV) during electrical stimulation to record evoked synaptic currents. To isolate GABA_A_-receptor mediated IPSCs, 10 μ M DNQX, 25 μ M DL-AP5, and 2 μ M CGP 52432 were used to block glutamatergic and GABA_B_-receptor mediated responses. Consistent with our other recordings, cells were excluded from analysis if the spikes failed to overshoot 0 mV (before junction potential correction) or access resistance exceeded 30 MΩ at any point during the experiment. We determined that the IPSCs reversed around −85 mV (junction potential corrected).

### Model development

In this section, we first describe how we derive our intrinsic and synaptic properties from our experimental recordings. Then, we describe the mathematical structure of our single PV+ cell model, as well as our network model. The model structure alone is described here, whereas specific parameter choices are described in the Results section. Finally, we define how we measure population frequency and coherent firing activity.

#### Intrinsic properties

The intrinsic properties of the PV+ interneurons were determined from whole-cell patch-clamp recordings of seven PV+ cells during the application of synaptic blockers. While the membrane potential of the cell was held at −60 mV in current clamp, a series of small-amplitude 1-s hyperpolarizing steps (10-pA increments) were used to determine the input resistance, *R*_in_ (MΩ), and membrane time constant, τ_*m*_ (ms). The input resistance was calculated by computing the slope of the voltage change over the amplitude of the current injected. The membrane time constant was derived by fitting the voltage change during a small hyperpolarizing current step with a single exponential function and calculating the mean fit over a few small current steps. The membrane time constant effectively represented the amount of time required for the membrane potential to reach ~63% of the total change. The capacitance was determined by τ_*m*_/*R*_*in*_. The action potential threshold was set to be the first voltage point such that the slope of the membrane potential exceeded 20 mV/ms (Bekkers and Delaney, 2001). The spike width was determined at the threshold value. In addition, the spike height and the minimum membrane potential reached following the spike were found. Fast-firing PV+ cells were identified as those that generated high-frequency trains (maximal frequencies greater than 100 Hz) of action potentials during depolarizing current pulses.

The f-I profiles of the PV+ cells are important to characterize, as we aim for our single cell model to respond to a variety of synaptic input strengths with frequencies similar to that observed experimentally. These f-I curves were determined by applying depolarizing current steps of 1 s duration to cells held in current clamp. Amplitudes were increased incrementally with step sizes of 50 pA for four of seven cells, and 10 pA for three of seven cells. The frequency (Hz) was determined as the inverse of the average inter-spike interval (ms) over the course of the 1 s step. For each cell where data was available, the approximate linear slope of the f-I curve above 60, 70, or 80 Hz was determined using a least squares method. These values were chosen since above 60–80 Hz the slope was well-approximated by linearization. In addition, the minimum amount of current required to initiate a spike, the rheobase current (*I*_rheo_, in pA), was determined.

To find the amount of spike frequency adaptation (SFA), we plotted the inter-spike interval (ISI) with respect to the latency of each interval from the start of the current step. The slope of the line fit to these points was used to quantify the amount of SFA (Hemond et al., 2008).

#### Synaptic constraints during population activities

**Figure 1:**
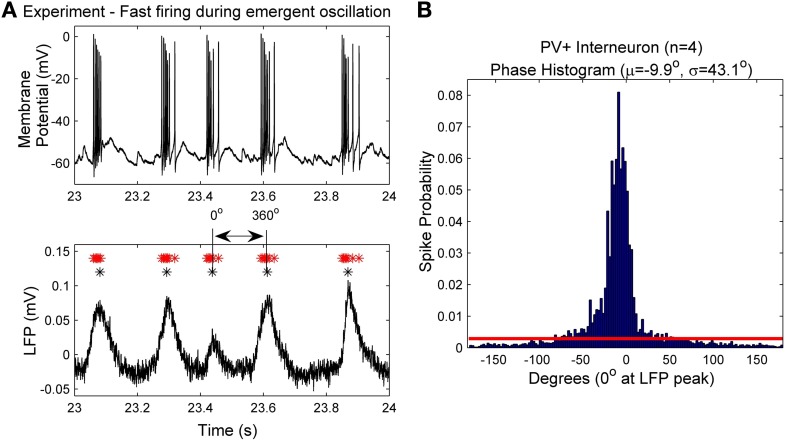
**(A)** An example intracellular recording of a PV+ interneuron's firing (top) during the emergent network rhythm (bottom). The peak times of the LFP are denoted with black asterisks (^*^), and the spike times are denoted with red asterisks (^*^). Each LFP peak is given a phase value of 0°, and the waveform based phase was determined by interpolating between the peaks. **(B)** The spike phases determined from the four PV+ interneurons with respect to the LFP peak (0°). Note the narrow window of phase in which the cells spike (mean and standard deviation given by μ and σ respectively). The red line denotes the cut-off value used to determine intra-cycle firing (−80° to 80°), which encompasses more than 90% of the spikes.

In order to estimate the amount of synaptic input a single PV+ cell receives during a period of high frequency firing, we consider the context of emergent oscillations in the intact hippocampal preparation. We noted that our LFP recordings have distinct periods of activity and of quiescence, and this allowed us to estimate the amount of synaptic input during the fast intra-cycle firing. To define the region in which the firing occurred, we used four extracellular recordings, and defined the peaks of the LFP as 0° (Figure 1A). We obtained the waveform-based phase by interpolating phase values between these peaks. The phase of each intracellular spike during the rhythm was then based on this interpolation. This allowed us to generate a spike-phase histogram from which we could identify the phase ranges in which the majority of spiking occurred. For the PV+ fast-spiking cells, the majority of spiking occurred in a relatively narrow region surrounding the 0° LFP peak (Figure 1B). Therefore, we defined an intra-cycle spike to be one that fell between −80° and 80° of the 0° LFP peak, as this comprised the vast majority of spikes (>90%), and excluded those that were spontaneously generated during the quiescent period of the oscillation. We defined the average frequency of intra-cycle firing as the mean of the inverse of the intra-cycle ISIs. We tried different phase ranges (e.g., −100° to 100°, −60° to 40°) and found that this did not have much of an effect on the intra-cycle firing frequencies, because the spiking activity is tightly phase-locked to the peaks of the LFP. We used these extracted fast firing frequencies in combination with our developed cellular model to estimate the synaptic drive (*I*_applied_) that PV+ fast-firing cells would receive during ongoing population activities. The precise estimate values of this *I*_applied_ parameter are determined in the Results section.

#### Single cell model structure

We built a simple model of a fast-firing PV+ interneuron using Izhikevich's (2003) two dimensional system of ordinary differential equations. It captures the subthreshold behavior of the interneuron and the upstroke of the action potential, using a reset mechanism to represent the spike's fast downstroke. An important advantage of this model is that it is relatively simple, but still allows us to choose parameters that are related to biophysical quantities (Izhikevich, 2003).

The Izhikevich (2003) model structure has a fast variable representing the membrane potential, *V* (*m*V), and a variable for the slow “recovery” current, *u* (pA). We used a slight modification of the Izhikevich model in order to reproduce the narrow spike width which is characteristic of these fast-firing PV+ interneurons. The model is given by:

(1) $$\begin{array}{l} \;C_{m}\dot{V}=k\text{​}\left(V-v_{r}\right)\left(V-v_{t}\right)-u-I_{\text{syn}}+I_{\text{applied}}\\ \;\dot{u}=a[b\text{​}\left(V-v_{r}\right)-u]\\ \text{ If }V\geq v_{\text{peak}},\text{then }V\leftarrow c,u\leftarrow u+d\\ \text{Where }k=k_{\text{low}}\text{ if }V\leq v_{t}k=k_{\text{high}}\text{ if }V>v_{t} \end{array}$$

The parameters are as follows:

*C*_*m*_ (pF) is the membrane capacitance.

*v*_*r*_ (mV) is the resting membrane potential.

*v*_*t*_ (mV) is the instantaneous threshold potential.

*v*_peak_ (mV) is the spike cut-off value.

*I*_syn_ (pA) represents the recurrent inhibition between PV+ cells.

*I*_applied_ (pA) is the applied current, and represents all other synaptic input to the PV+ cell.

*a* (ms^−1^) is the recovery time constant of the adaptation current.

*b* (nS) describes the sensitivity of the adaptation current to subthreshold fluctuations. Greater values couple *V* and *u* more strongly resulting in possible subthreshold oscillations and low-threshold spiking dynamics. Further, the sign of *b* determines whether the effect of *u* is amplifying (*b* < 0) or resonant (*b* > 0).

*c* (mV) is the voltage reset value.

*d* (pA) is the total amount of outward minus inward currents activated during the spike and affecting the after-spike behavior.

*k* (nS/mV) represents a scaling factor. *k*_high_ is used to adjust the spike width after the threshold, to allow the for the narrow spike width of fast-firing PV+ interneurons to be appropriately represented.

The parameters *v*_*r*_, *v*_*t*_, *v*_peak_, and *c* were directly based on the intrinsic spike characteristics derived from recordings of PV+ interneurons. Specifically, using the average results over the seven recorded cells, *v*_*r*_, *v*_*t*_, *v*_peak_, and *c* were set to represent the mean resting membrane potential, threshold potential, peak height, and voltage reset value, respectively. *k*_high_ was determined such that the width of the action potential from threshold in the model matched the average spike width at threshold in the biological cells. The adaptation parameters *a* and *d* were set such that the cell model does not exhibit SFA, in accordance with the minimal adaptation recorded from our cells (see Results). Finally, the parameters *b* and *k*_low_ were chosen such that the slope of the model f-I profile (f-I curve) was within the range of slopes determined from the experimental f-I curves, and the rheobase current of the model was equal to the average experimental rheobase current.

We varied *b* and *k*_low_, to produce multiple models and determined the rheobase current of each model, as well as the slope of each model's f-I curve over 60 Hz (using a least squares approach). We explored the parameter ranges such that *k*_low_ was varied from 0 to 20 and *b* was varied from −10 to −0.1 (each with a step size of 0.1). To confirm that this cut-off of 60 Hz was appropriate, we also determined the slope above 70 and 80 Hz (as was done for the experimental data), but found little difference. These values of *b* and *k*_low_ were chosen while maintaining a model rheobase at the physiologically determined average rheobase. The frequency values above which we fit were chosen due to the shape of the model's f-I curve: it becomes steeper as the input current decreases, and more gradual as it increases. Therefore, a linear fit of the whole f-I curve would not be appropriate, but is reasonable for the gradual curve greater than 60 Hz. In this way, when the parameters of this simple model are fit, the model has direct ties with the experimental data.

#### Network model structure

We built network models using our developed models of individual PV+ fast-firing interneurons. Here, we describe how we constrained our synaptic parameters using experimentally determined values for: the inhibitory reversal potential, the synaptic kinetics, the synaptic conductance strengths, and the overall synaptic drive. We then describe size and connectivity constraints used for our networks.

First, we used inhibitory reversal potentials as determined from our experiments (see above) in our model of the PV-PV synaptic connections [Equation (2)]. Therefore, each model cell received PV-PV inhibitory input through a chemical synapse represented by:

(2) $$I_{\text{syn}}=g_{\text{syn}}s(V-E_{\text{syn}})$$

where *g*_syn_ (nS) is the maximal synaptic conductance, and *E*_syn_ = −85 mV is the inhibitory reversal potential.

The gating variable, *s*, represents the fraction of open inhibitory synaptic channels, and is given by first order kinetics (Destexhe et al., 1998; Ermentrout and Terman, 2010):

(3) $$\dot{s}=\alpha \left[T\right](1-s)-\beta s$$

The time course of unitary IPSCs has been measured in paired recordings of basket cells in CA1 hippocampal slices (Bartos et al., 2002). Specifically, rise and decay time constants were found to be 0.27 and 1.8 ms, respectively. The parameters α and β in Equation (3) represent the inverse of the rise and decay time constants, respectively (ms^−1^). [T] represents the concentration of transmitter released by a presynaptic spike. Supposing that the time of a spike is *t* = *t*_0_, then [T] is represented by a unitary pulse, lasting for 1 ms (until *t*_1_). Then, we can represent

(4) $$s\text{​}\left(t-t_{0}\right)=s_{\infty }+\left(s(t_{0})-s_{\infty }\right)\text{​}e^{-\frac{t-t_{0}}{\tau_{s}}},t_{0}<t<t_{1}$$

where

(5) $$s_{\infty }=\frac{\alpha }{\alpha +\beta }\text{ and }\tau_{s}=\frac{1}{\alpha +\beta }$$

After the pulse of transmitter has gone, *s*(*t*) decays as

(6) $$s(t)=s\left(t_{1}\right)e^{-\beta (t-t_{1})}$$

Second, from their network models and experimental constraints, Bartos et al. (2002) estimated a unitary postsynaptic peak conductance density of 0.04 ms/cm^2^. This value was based on a recorded peak current of 208 pA and a somatodendritic surface area of ~12,000 μm^2^ (Bartos et al., 2001). Using this same surface area, this translates to a unitary postsynaptic peak conductance of 4.8 nS. We examined a range of *g*_syn_ from 0 to 10 nS in order to investigate the behavior of the network in the parameter regime surrounding the experimentally determined peak value of 4.8 nS.

Third, while we explicitly modeled the synaptic input from each PV+ cell [Equation (2)], we represented all other synaptic input to the PV+ cells through an applied current (*I*_applied_). These applied currents (in pA) were constant input to individual cells, and both homogeneous and heterogeneous *I*_applied_ values were considered across the population of cells. The values of *I*_applied_ were based on estimates of synaptic input to PV+ interneurons during the emergent network rhythm (see section Synaptic Constraints During Population Activities).

In the work here, we used appropriately sized and connected inhibitory networks, and a wide range of maximal synaptic conductance (*g*_syn_) and synaptic drive (*I*_applied_) parameter values to determine the conditions under which our model networks could exhibit coherent firing.

**Figure 2:**
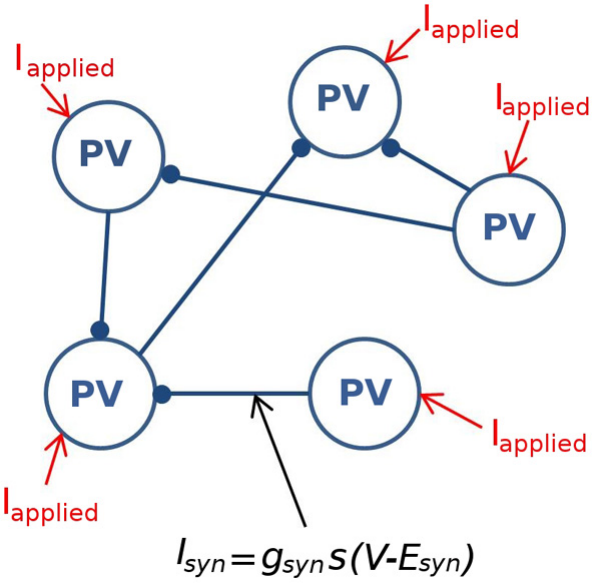
**A schematic of the fast-firing PV+ interneuron network.** The network is composed of 500 PV+ cells, each connected with ~60 other PV+ cell models. The PV-PV inhibitory connections are represented by *I*_syn_, all other synaptic drives are represented with *I*_applied_.

***Network size and connectivity***Importantly, we need to consider an appropriate network size and connectivity. In other words, what amount of physical space should our model represent, and how many individual neuron models are required to represent this space? Answers to these questions are non-trivial but need to be clearly considered so that our experimentally constrained values are applied appropriately to our models. In the experimental setting of the intact hippocampal preparation producing its own rhythm, Sik et al. (1995) investigated the CA1 interneuronal network of PV+ basket cells *in vivo* and determined that in the stratum pyramidale a single PV+ basket cell makes synaptic contacts with at least 60 other PV+ cells in a spatial region of the volume up to 0.1–0.2 mm^3^. Because we only know connectivity in the stratum pyramidale, we design our model network to encompass that region as a representative of the experimental setting. Since the density of PV+ cells in the pyramidal layer is 3.83–5.73 × 10^3^ cells/mm^3^ (Aika et al., 1994; Jinno and Kosaka, 2006), this volume of tissue comprises ~380–1150 PV+ cells. Therefore, we create network models that are composed of 500 individual fast-firing PV+ cells models. The network is randomly connected with a probability of 0.12, so that each cell is connected to ~60 other PV+ cells. A schematic of this network is given in Figure 2.

#### Measuring population frequency and coherent firing activity

Population activity was taken to be the average membrane potential of all model cells in the inhibitory networks. While this does not directly represent the LFP, it can be considered as a gross approximation given the following: since the largest membrane potential averages would correspond to the most inhibitory cells spiking closely together, this imposes the most inhibitory synaptic currents onto “output cells,” producing a representative population output. We note that average membrane potentials have been used in other network models to represent population activity—e.g., see Bähner et al. (2011). We ran additional simulations where the population activity was instead represented by an average of the summed synaptic variables (∑_*i*_s_*i*_/*N*), and the main results were unchanged.

Using the fast Fourier transform, the network frequency is defined as the frequency at which there is a spectral peak in the population activity in the last 500 ms of our 1.5 s simulations. We note that obtaining the network frequency in this way does not mean that there is also coherent firing activity in the network: additional measures are required.

To determine the amount of coherent firing that the network exhibits, we used a measure based on the normalized cross-correlations of neuron pairs in the network (Gerstein and Kiang, 1960; Welsh et al., 1995; Wang and Buzsáki, 1996). Consider two neurons, *i* and *j*, firing within a given time bin τ. Then their respective spike trains are given by *X(l)* = 0,1; *Y(l)* = 0,1 where *l* = 1, 2, … *K*, and the total amount of time is given by *T* = τ*K*. The amount of coherent firing between the two neurons is given as the cross-correlation between these two spike trains:

(7) $$\varphi_{ij}=\frac{\sum_{l\;=\;1}^{K}X(l)Y(l)}{\sqrt{\sum_{l\;=\;1}^{K}X(l)\overset{K}{\underset{l\;=\;1}{\sum }}Y(l)}}$$

φ is given as the average of φ_*ij*_ over all neuron pairs in the network, and we refer to it as the population coherent firing measure. φ is between 0 and 1, and is close to 1 for maximal synchrony, and close to 0 for maximal asynchrony. We used τ = 0.1/(network frequency), and we calculated the average population firing coherence during the last 500 ms of our 1.5 s simulations (φ_*avg*_). We performed additional simulations with τ = 1 ms, and our main results were unchanged.

To determine the ranges of synaptic conductance values (*g*_syn_) and applied input (*I*_applied_) (synaptic drive) for which our network exhibited coherent firing, we define a “synchronized network” as one in which the average population coherent firing measure over the last 500 ms of our simulation is greater than or equal to 0.2 (φ_*avg*_ ≥ 0.2). We chose 0.2 as a cut-off, since almost all population coherent measures were either well below or were above this value.

**Figure 3:**
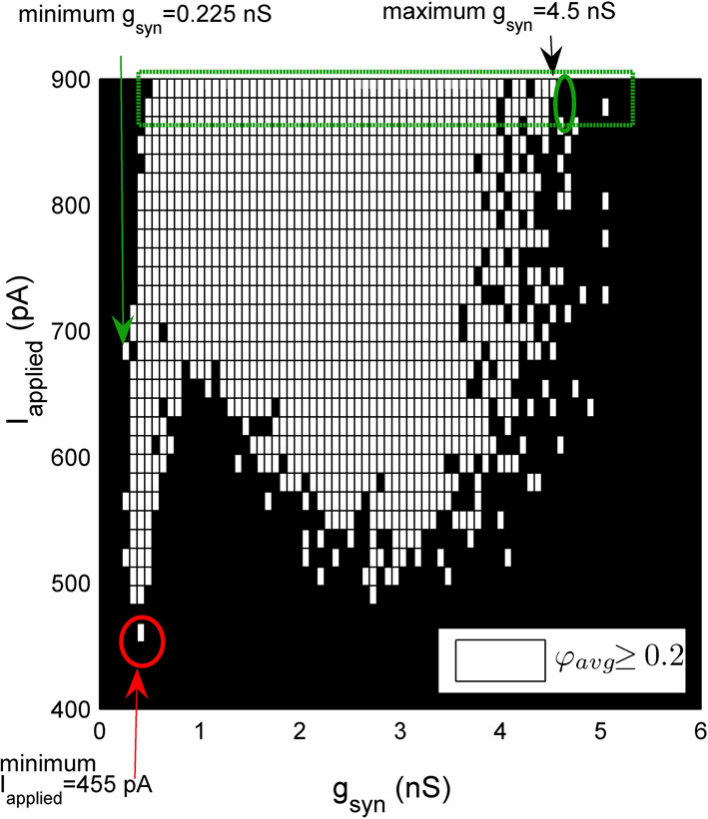
**A schematic demonstrating the method used to quantify the window of robust coherent firing over *g*_syn_ and *I*_applied_.** In this heterogeneous network example, *I*_applied_ has a Gaussian distribution with its standard deviation equal to 12 pA. 500-cell network simulations demonstrating coherence (average population coherent firing measure φ_avg_ ≥ 0.2) are shown in white, and those with non-coherent firing (φ_avg_ <0.2) are shown in black for a range of *g*_syn_ and *I*_applied_ values. The minimum *g*_syn_ value is defined as the smallest *g*_syn_ such that the network demonstrates coherence (minimum *g*_syn_ = 0.225 nS, green arrow for example shown here). To determine the maximal *g*_syn_ for which our network exhibited robust coherent firing, we considered the last two rows of *I*_applied_ values (green rectangle). Once coherence began (~ *g*_syn_ = 0.525 nS), we consider increasing values of *g*_syn_ for these last two rows of *I*_applied_. The first instance where both rows of *I*_applied_ are non-coherent (green ellipse), we know that our *g*_syn_ has surpassed its maximum. Thus, maximal *g*_syn_ is given by the preceding step (maximal *g*_syn_ = 4.5 nS for example shown here, black arrow). Our minimal *I*_applied_ value is the minimal *I*_applied_ such that our networks produced coherent firing (red arrow).

To identify boundaries on our region of coherence, we did a simple quantification algorithm that is described in detail in Figure 3. First, the minimal *g*_syn_ was defined as the smallest *g*_syn_ such that the network demonstrated coherence (φ_*avg*_ ≥ 0.2) (green arrow, Figure 3). To identify the maximal *g*_syn_ value we considered the last two rows of *I*_applied_ values (dashed green rectangle, Figure 3). Once coherence began, we consider increasing values of *g*_syn_ for these rows. Our maximal *g*_syn_ (black arrow, Figure 3) is the step preceding the point where both rows of *I*_applied_ are non-coherent (green circle, Figure 3). The minimal *I*_applied_ was defined as the minimal *I*_applied_ such that our networks produced coherent firing (red arrow, Figure 3).

### Simulations

Our experimental data analysis is done using custom codes created in MATLAB. Network model runs, coherent firing, and population activity determinations were done using the Brian simulator (Goodman and Brette, 2009). The initial conditions of our membrane potentials (V) were chosen to be uniform random values from −55 to −65 mV. We used the forward Euler method for integration with a time step of 0.001–0.01 ms. Our simulations were run on the GPC supercomputer at the SciNet High Performance Computing Consortium (Loken et al., 2010) (http://www.scinethpc.ca/).

## Results

Under what conditions can networks of CA1 PV+ fast-firing interneurons directly contribute to the generation of fast, population rhythms? We address this question using a mathematical modeling approach that takes advantage of an intact *in vitro* hippocampal preparation that spontaneously exhibits population rhythms. As detailed below, our network models are closely intertwined with experimental data at multiple levels from the same experimental situation. By constraining our model parameter values with directly measured experimental values, our PV+ interneuron cell and network models reflect physiology with greater specificity and accuracy in comparison with existing fast-firing interneuron models. Using simulations, we vary inhibitory synaptic strengths (*g*_syn_) and overall synaptic drive (*I*_applied_) to determine the conditions under which our networks exhibit coherent firing activities. Our results indicate that while our networks can produce coherent firing at high frequencies, they do so only for relatively small inhibitory conductance strengths (compared with the prevailing literature), and large excitatory drives (compared with our experimentally determined values). These constrained networks exhibit a sharp transition from random to coherent firing. Even with only a small change in synaptic input, many more cells are recruited into the coherent state. This may indicate a design property of fast-spiking interneuron networks to allow the generation of theta/high frequency network oscillations.

### The experimentally constrained individual ca1 PV+ interneuron cellular model

To create a fast-firing PV+ interneuron model, we first recorded the activity of this interneuron subtype in the intact hippocampal preparation (see Methods for details). The intrinsic properties of the seven recorded PV+ interneurons, determined in the presence of synaptic blockers, demonstrated fast-firing (>100 Hz), narrow spike widths (from threshold: 0.83 ± 0.12 ms; mean ± S.E.M.), and short time constants (5.87 ± 1.04 ms). They were determined to have an input resistance of 73.29 ± 12.74 MΩ which, in combination with the short time constant, gave membrane capacitances of 81.14 ± 19.4 pF. These values are in agreement with previous fast-firing PV+ interneuron recordings (Bartos et al., 2001). The spike shape of the PV+ interneuron action potentials were determined on the basis of resting membrane potential (−60.6 ± 3.3 mV), threshold membrane potential (−43.1 ± 4.2 mV), maximal spike height (2.5 ± 4.3 mV), spike width at threshold (0.83 ± 0.12 ms), and minimal membrane potential (−67.0 ± 2.4 mV). The rheobase current is defined as the minimal amount of current required to elicit a spike. We used a series of depolarizing 10 pA steps, as done in three of the seven cells, to precisely determine the rheobase current (131 ± 58 pA). The values for all these parameters are summarized in Table 1. In this way, our model is constrained with experimentally determined intrinsic properties.

**Table 1:** **Experimental values compared with model parameters**.

**Parameter**	**Experiment (*n* = **7**)**	**Model**
*v*_r_ (mV)	−60.6 ± 3.3	−60.6
*v*_t_ (mV)	−43.1 ± 4.2	−43.1
*v*_peak_ (mV)	2.5 ± 4.3	2.5
*c* (mV)	−67.0 ± 2.4	−67
*C*_m_ (pF)	81.14 ± 19.4	90
*I*_rheo_ (pA)	131 ± 58 (*n* = 3)	131

From top down, parameters are: resting membrane potential, threshold potential, action potential peak, after-hyperpolarizing potential minimum, membrane capacitance, and rheobase current. Experimental values are given as mean ± S.E.M.

**Figure 4:**
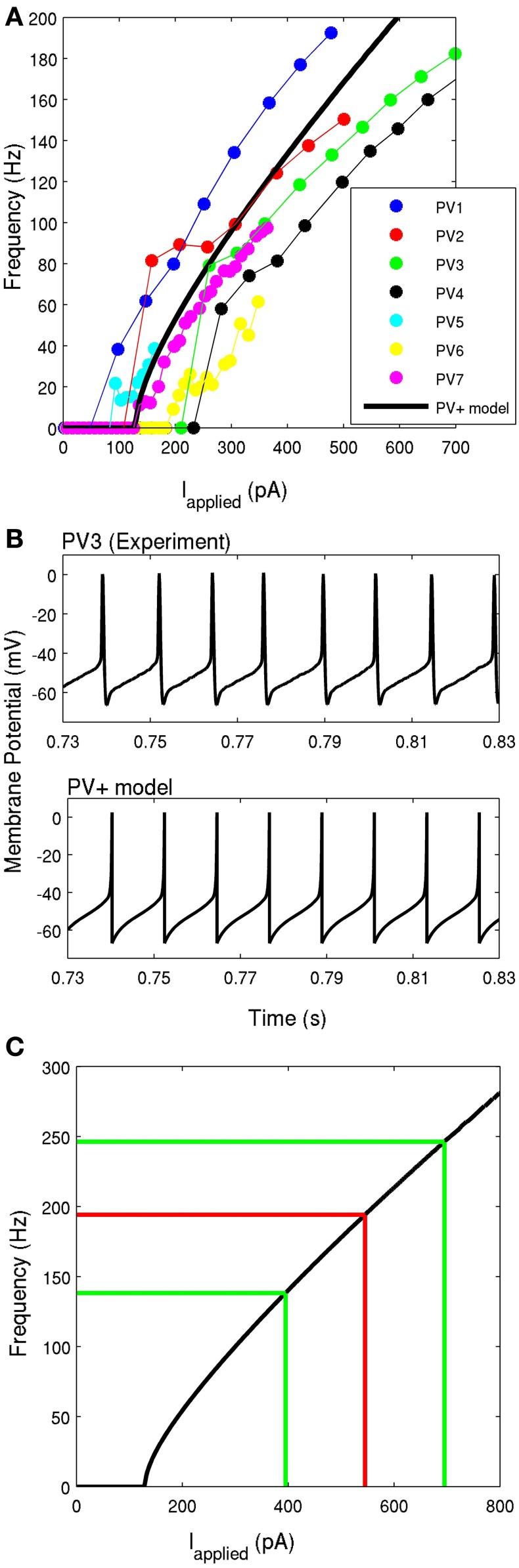
**(A)** The frequency-current profiles of the four recorded PV+ interneurons, determined under current clamp with steps of 50 pA for four PV+ cells (represented by blue, red, green, and black), and steps of 10 pA for three PV+ cells (represented by cyan, yellow, and purple). Note that the solid points denotes data points, lines in respective colors denote estimated values. The PV+ cell model's f-I curve is given in a thick black solid line, where the slope of the curve and the rheobase current is within the experimental range. **(B)** An example intracellular recording of a PV+ cell during current clamp with applied current of ~260 pA (top) is compared with the firing of our PV+ cell model with an applied current of 260 pA (bottom). The spike characteristics and firing rates of the model closely match those of the experiment. **(C)** The frequency-current profile of the PV+ interneuron model is shown in black. The mean frequency of intra-theta-cycle firing of PV+ interneurons during the network rhythm is shown in red. The mean input current is determined based on the f-I curve. The green lines indicate one standard deviation from the mean current (*n* = 4).

It is important to also constrain the f-I profile with experimental data, so that the amount of synaptic input the cell model receives will directly correlate to the appropriate spike frequency of the cell. The average frequencies of each cell were plotted as a function of the amount of depolarizing current, and are given in Figure 4A. The approximate slopes of these f-I curves were used to constrain the model f-I curve. To determine the SFA, we plotted the ISI with respect to the latency of each interval from the start of the current step, and fit the slope of the line to these points (Hemond et al., 2008). This slope was determined to be 0.0151 ± 0.0091, which quantifies the very minimal adaptation observed. Therefore, consistent with previous studies (Pawelzik et al., 2002), we found that CA1 PV+ interneurons are fast-firing neurons with narrow spike widths and little SFA.

Using the mathematical model structure given in the Methods, the resting membrane potential, threshold potential, maximal spike height, and membrane potential minimum were fit directly with the experimentally determined values, as summarized in Table 1. That is, we set *v*_*r*_ =−60.6 mV, *v*_*t*_ = −43.1 mV, *v*_peak_ = 2.5 mV, *c* = −67 mV. The parameter *k*_high_ = 14 nS/mV is a scaling factor that allowed us to adjust the spike width above the threshold, to appropriately represent the narrow spike width of our PV+ interneurons. The values *a* and *d* were chosen such that our model does not exhibit SFA, which is representative of the lack of adaptation expressed by these cells. They are given by *a* = 0.1 ms^−1^, *d* = 0.1 pA.

Next, we determined the parameters *k*_low_ and *b*. Since our interneurons showed little adaptation or sensitivity to subthreshold oscillations, we were restricted to *b* <0 (Izhikevich, 2003). To maintain consistency with our experimental findings, we chose *k*_low_ and *b* such that: (1) our model rheobase current was equal to the average experimental rheobase current (experiment: *I*_rheo_ = 131± 58 pA, model: *I*_rheo_ = 131 pA), and (2) the slope of the model f-I curve over 60, 70, or 80 Hz was in the range of those determined experimentally (as described in Methods). We determined that our best fit to maintain these properties was with *k*_low_ = 1.7nS/mV and *b* =−0.1 nS. The membrane capacitance affects the f-I profile of the cell, and it was set within its physiological range to *C*_*m*_ = 90 pF.

In Figure 4B, the model is shown in comparison to an example experimental recording, both with an applied current of 260 pA. It is evident that the spike shape and firing properties are consistent with our experimental findings. The model f-I curve is shown in comparison with the experimental curves for the seven recorded cells in Figure 4A, and on its own in Figure 4C. The comparison of model parameters with experimental values is given in Table 1.

To our knowledge, this is the first simple, single compartment model for CA1 PV+ fast-firing interneurons that has been tied directly with experimental data in several ways and which faithfully simulates the firing behavior and spike characteristics of CA1 PV+ interneurons. As such, it provides a useful platform with which one can investigate the role of PV+ fast-firing interneurons in a variety of behaviors.

### The experimentally constrained ca1 PV+ interneuron network model

During emergent theta oscillations in our intact hippocampal preparation *in vitro*, PV+ interneurons in the CA1 region spike heavily within each theta cycle as shown for one of the experimental recordings in Figure 1A. We note that high frequency oscillations (HFOs; >100 Hz) are recorded in the CA1 region of the hippocampus, and are often nested in theta oscillations (3–12 Hz) (Scheffer-Teixeira et al., 2012). In addition, Jackson et al. (2011) demonstrated that the subiculum of the intact hippocampal preparation spontaneously generates high frequency rhythms, which they referred to as fast gamma (100–150 Hz), and these rhythms are co-expressed with theta rhythms (3–10 Hz). These fast network rhythms were abolished with fast-GABAergic blockers, but not by blocking AMPA/kainate or NMDA receptors, indicating an inhibitory network is responsible for these rhythms (Jackson et al., 2011). Networks of fast-firing interneurons have been shown in models (Wang and Rinzel, 1992; Traub et al., 1996; Wang and Buzsáki, 1996; White et al., 1998, 2000; Bartos et al., 2001) to produce gamma (30–100 Hz) oscillations. Therefore, we consider our network model in the following way: by constraining our synaptic drive with the total synaptic input to PV+ interneurons during emergent theta oscillations, do we obtain synchronized network outputs (see Methods for details) at high frequencies?

#### Synaptic drive estimated based on periods of high frequency firing during theta oscillations

In order to determine the amount of excitatory drive that each cell receives during these periods of high frequency firing, we note that PV+ interneurons in general receive mainly excitatory input, and comparatively much less recurrent inhibition (Gulyás et al., 1999). For example, in PV+ basket cells, which comprise the largest population (~60%) of PV+ interneurons (Baude et al., 2007), ~94% of the synaptic connections they receive are excitatory, whereas only ~2% come from other PV+ interneurons (Gulyás et al., 1999). Thus, during our emergent theta network rhythm, it is reasonable to assume that the majority of synaptic input to the PV+ interneurons is from sources other than inhibition from other PV+ interneurons. With this assumption, we constrain the drive to each interneuron model in the network by approximating the total amount of input received during each theta oscillation.

Since the theta oscillations exhibit distinct periods of activity and quiescence, as correlated with PV+ cell firing, we can estimate the amount of synaptic input received during the “activity” period. As described in the Methods, we use the peaks (at 0°) of our extracellular LFP recordings (Figure 1A, black asterisks) to obtain the phase of the peak of each action potential (Figure 1A, red asterisks). From the resulting spike-phase histogram (Figure 1B), and defining an intra-cycle spike to be one that fell between −80° and +80° of the LFP peak, we obtain PV+ firing frequencies that exist during the emergent theta oscillations. These frequencies for our four recordings are 179.6 ± 3.4, 210.7 ± 85.3, 153.4 ± 79.0, and 192.4 ± 88.0 Hz. Using these frequencies in combination with our model f-I curve (Figure 4C) we infer the mean amount of synaptic current that was present during the network rhythm (545 ± 150 pA). The approximate standard deviations are shown as the green lines, and the mean in red, in Figure 4C. We used these experimentally determined ranges of synaptic input during the fast-firing stage of each theta cycle to constrain our synaptic drive to each cell model (*I*_applied_).

We now use our experimentally constrained cellular and network models to investigate the conditions under which high frequency population rhythms could occur. Specifically, we examine a wide range of maximal synaptic conductance (*g*_syn_) and synaptic drive (*I*_applied_) parameter values that incorporate physiological constraints to determine the conditions under which our networks could exhibit coherent firing. We chose to examine a large range of excitatory *I*_applied_ from 200 to 900 pA (using a step sizes of 10 or 15 pA), which fully encompasses the physiological range of 545 ± 150 pA determined above. In addition, we varied the maximal synaptic inhibitory conductances, *g*_syn_, from 0 to 10 nS (using a step size of 0.05 or 0.075 nS), which also fully encompasses the physiological range (see Methods). For each combination of these two parameters, we determined the population frequency and population coherent firing measure of the network, and networks were deemed to be “synchronized” if they exhibited a population coherent firing measure greater than or equal to 0.2 (see Methods).

#### High frequency population rhythms occur in model networks when inhibitory synaptic strengths are relatively small and excitatory drive is relatively large

**Figure 5:**
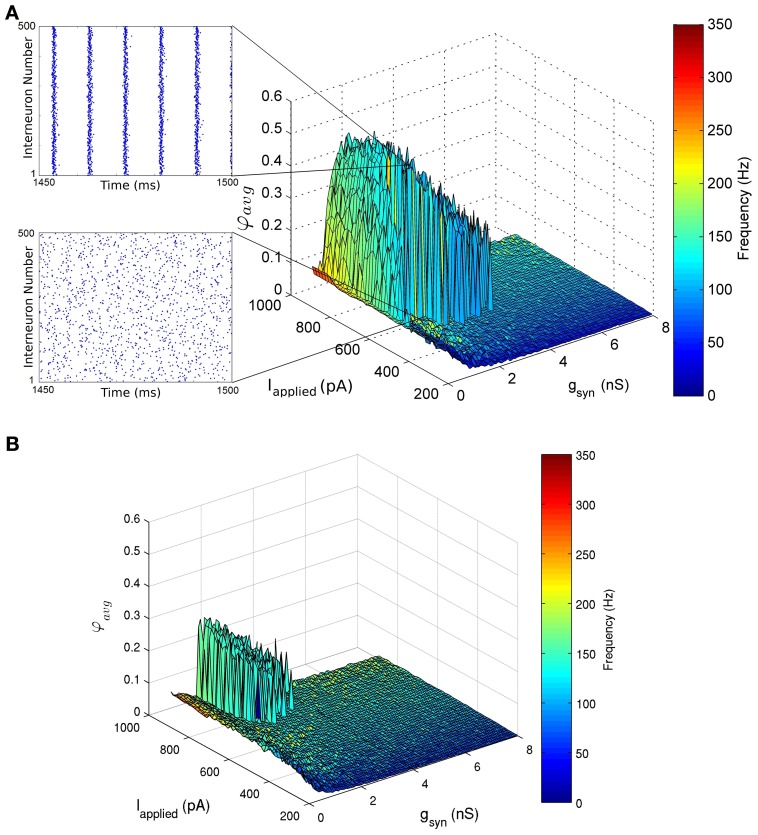
**Network coherence measures for our 500 PV+ cell network with heterogeneous external synaptic drive (*I*_applied_), over a range of applied currents and peak conductance values (*g*_syn_).** Each network is randomly connected with a 0.12 probability of connections (in accordance with estimates from Sik et al. (1995), where PV+ interneurons were estimated to contact with 60 other PV+ interneurons). The color bar on the right represents the frequency of the network oscillation. **(A)** The standard deviation of the applied current is set to 12 pA. This experimentally constrained network exhibited coherent fast rhythms (90–197 Hz) in the high frequency oscillation (HFO) range (~120–160 Hz) (where coherent networks were defined as those with an average population coherent firing measure φ_*avg*_ ≥ 0.2). This coherent firing was found for a range of conductance strengths 0.225 nS ≤ *g*_syn_ ≤ 4.5 nS and tonic excitatory input *I*_applied_ ≥ 485 pA. Raster plots of the last 50 ms of simulation are shown as insets. One network shows no coherence (φ _avg_ = 0.05) for *I*_applied_ = 595 pA, *g*_syn_ = 1.5 nS (bottom). The neighboring network exhibits coherence (φ _avg_ = 0.51) for *I*_applied_ = 600 pA, *g*_syn_ = 1.5 nS (top). **(B)** A heterogeneous network with the standard deviation of *I*_applied_ equal to 50 pA. The network exhibits coherent high frequency rhythms (129–163 Hz for φ _avg_ ≥ 0.2) for 1.125 nS ≤ *g*_syn_ ≤ 2.4 nS and *I*_applied_ ≥ 710 pA.

We investigated whether high frequency (>100 Hz) population rhythms occur for our chosen ranges of synaptic drive (200 pA ≤ I_applied_ ≤ 900 pA) and inhibitory synaptic PV-PV conductance strengths (0 nS ≤ *g*_syn_ ≤ 10 nS), which encompass our experimentally determined ranges. To do so, we examined 500-cell networks where *I*_applied_ was homogeneous, as well as the more realistic situation in which the synaptic drive was heterogeneous across all cells. In particular, heterogeneous input across the network was given by normally distributing inputs with a mean of *I*_applied_ and standard deviation of 5, 12, 25, 50, or 75 pA. As expected from previous modeling studies (Wang and Buzsáki, 1996), coherent firing was sensitive to heterogeneity (compare network simulations with the standard deviation of *I*_applied_ equal to 12 and 50 pA in Figures 5A,B, respectively).

The networks produced coherent population firing, but did so with population frequencies that were necessarily fast (Figure 5). For example, in the network where *I*_applied_ had a standard deviation of 12 pA, coherent firing was produced for population frequencies of 90–197 Hz for the parameter ranges examined (Figure 5A). Although such high frequency population rhythms were produced in our CA1 network models, they did so for relatively small inhibitory conductance strengths (*g*_syn_) compared with estimates derived from Bartos et al. (2002) (4.8 ± 1.2 nS), and large excitatory drives (*I*_applied_) as compared to our experimentally determined parameter ranges (545 ± 150 pA). For example, in the heterogeneous network where *I*_applied_ had a standard deviation of 12 pA, high frequency population rhythms were robust (see Methods for details) for 0.225 nS ≤ *g*_syn_ ≤ 4.5 nS and *I*_applied_ ≥ 455 pA (Figure 5A). Although the inhibitory conductance strengths (*g*_syn_) were small, they were necessarily greater than zero, demonstrating that PV-PV network connections are required to generate these fast rhythms.

As expected, the window in which coherent population firing is exhibited decreased with increased heterogeneity. In comparison, the heterogeneous network with a 50 pA standard deviation of *I*_applied_ produced robust coherent firing (between 129 and 163 Hz) for 1.125 nS≤ *g*_syn_ ≤ 2.4 nS and *I*_applied_ ≥ 710 pA (Figure 5B). Thus, in more heterogeneous networks, robust rhythms require larger *I*_applied_ values and a reduced range of possible *g*_syn_ values, where the maximal *g*_syn_ values are smaller and the minimal are larger. Of course, describing synaptic input as an applied current with heterogeneity is an approximation. To ensure that describing *I*_applied_ in this way is a reasonable choice, we also ran several simulations in which *I*_applied_ was represented as noisy excitatory and inhibitory synaptic currents as done in previous work (Ho et al., 2012). The results of these simulations were similar to the heterogeneous runs (not shown), although a full exploration of excitatory and inhibitory balances was not done at this time.

In conclusion, our inhibitory network needs enough excitatory drive to produce coherent high frequency firing, and connections between the inhibitory cells cannot be too strong. These results were maintained with various levels of heterogeneity, although the windows in which coherent firing was obtained reduced with increased heterogeneity. The prediction that a large excitatory drive to PV+ cells is required to produce coherent high frequency firing can be tested experimentally. For example, one could optogenetically stimulate (or silence) this cell population, and see if high frequency population oscillations are generated (or diminished).

#### Sharp transitions in and out of coherence depend on appropriate connectivity

An interesting feature produced by our network simulations is that the networks exhibited a sharp transition from random firing to network coherence with only a small change in *I*_applied_ (see raster plots in Figure 5A). Although the window of coherent activity was decreased as heterogeneity in the system was increased, this feature was maintained. Thus, small parameter perturbations, specifically to the excitatory drive, may allow the network to be easily gated in and out of coherent high frequency firing. PV+ interneurons are highly connected to neighboring pyramidal cells (Sik et al., 1995), and may have the potential to more strongly influence network oscillations during their coherent state. Therefore, this may provide a mechanism by which HFOs are nested in slower network rhythms.

**Figure 6:**
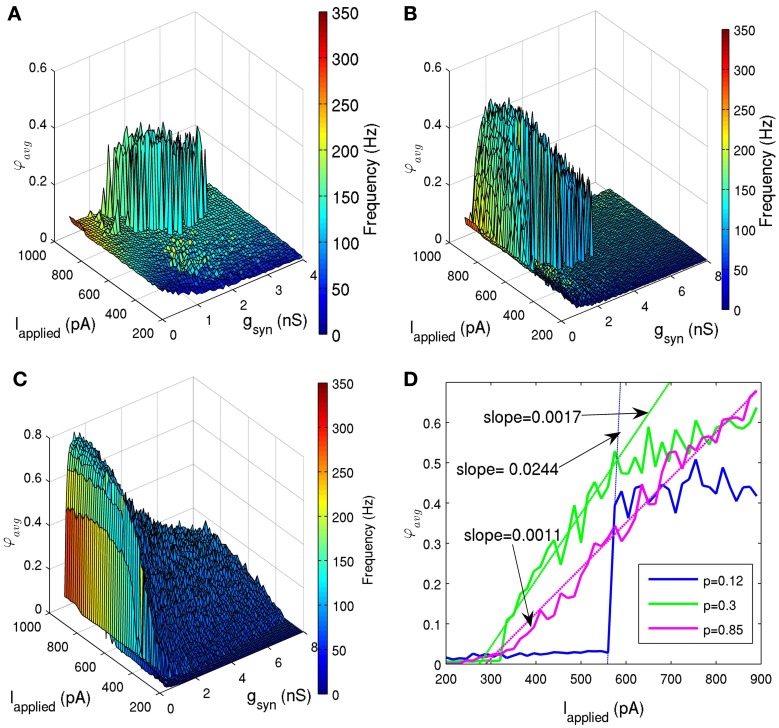
**(A,B,C)** Network coherence measures for our 500 PV+ cell network with heterogeneous external synaptic drive with the standard deviation of 12 pA, simulated over a range of applied currents (*I*_applied_) and peak conductance values (*g*_syn_). The color bar on the right represents the frequency of the network oscillation. **(A)** The connection probability was reduced from 0.12 [shown in **(B)**] to 0.08. The window of coherence (φ_*avg*_ ≥ 0.2) decreased dramatically, but the sharp transitions between random and coherent firing remained. **(B)** The connection probability is 0.12 (in accordance with our 500 cell network size, and estimates from Sik et al. (1995) where PV+ interneurons were estimated to contact with 60 other PV+ interneurons). Coherent firing (φ_*avg*_ ≥ 0.2) was found for a range of conductance strengths (0.225 nS ≤ *g*_syn_ ≤ 4.5 nS) and tonic excitatory input *I*_applied_ ≥ 485 pA). **(C)** The connection probability was increased from 0.12 to 0.5. The window of coherence (φ_*avg*_ ≥ 0.2) increased (0.075 nS ≤ *g*_syn_ ≤ 6.45 nS and *I*_applied_ ≥ 320 pA), but the transitions between random and coherent firing are gradual. **(D)** The network coherence measure is plotted against the amount of applied current (*I*_applied_) for networks with three different connection probabilities (solid lines in blue, green, and purple for connection probabilities, p, of 0.12, 0.3, and 0.85, respectively) and *g*_syn_ = 1.95 nS. The transitions were fit with lines (dashed lines), and the slopes were determined to quantify the steepness of these transitions. The experimentally determined connectivity of 0.12 (for a 500 cell network) (Sik et al., 1995) has a transition slope of 0.0244, which decreases in steepness as connectivity is increased (slope of 0.0017 for connectivity probability of 0.3, and slope of 0.0011 for connectivity probability of 0.85).

To determine how network connectivity would affect its ability to generate coherent firing, we held the standard deviation of *I*_applied_ at 12 pA, and ran simulations with various connection probabilities (*p* = 0.05, 0.06, 0.08, 0.12, 0.2, 0.3, 0.5, 0.85). Note that the biologically appropriate connection probability is about 0.12, since our determined network size was 500, and each cell connects with ~60 other cells (Sik et al., 1995). Figures 6A–C shows the effect of decreased connectivity (*p* = 0.08) and increased connectivity (*p* = 0.5), respectively. As connectivity was decreased, the sharp transitions between random and coherent firing remained (Figure 6A). However, the window of coherent firing decreased, and for very small connectivities (*p* ≤ 0.06), coherent firing was lost within our experimentally determined parameter ranges. As we increased connectivity in the network beyond experimentally estimated values, this sharp transition disappeared (Figures 6C,D). Instead, a larger window of coherence was achieved (e.g., for *p* = 0.5, 0.075 nS ≤ *g*_syn_ ≤ 6.45 nS and *I*_applied_ ≥ 320 pA), but with a smooth transition from random to coherent firing. This difference in transition from random to coherent firing was quantified in Figure 6D, where we considered a particular *g*_syn_ value, and demonstrated how the slope of the coherence measure with respect to *I*_applied_ decreased for increased connection probabilities. Therefore, network connectivity may not only affect its ability to produce coherent firing, but also may affect the mechanism by which this coherence is obtained. In addition, we explored various network sizes to see how it affects network coherence. We found that if we decreased the network size significantly, but maintained our connection probability (*p*), sharp transitions were lost. This serves as another illustration that network connectivity may change key features of the network output.

#### More cells are active during coherent firing

The smooth transitions are created due to the fact that the highly connected networks are able to fire coherently with smaller *I*_applied_ values. With a smaller excitatory drive, only a few cells fire, but do so coherently. As *I*_applied_ increases, gradually more cells are recruited into the rhythm, thereby increasing the coherence measure. In contrast, in our model networks with experimentally constrained connectivity, larger *I*_applied_ values are required to produce coherent activity. Therefore, a large number of cells, which are randomly firing, become coherent with only a small change in *I*_applied_.

To investigate how many cells are recruited into the coherent networks, we examined pairs of networks that lay directly on either side of the sharp transition. Thus, each pair of networks had one coherently firing network, and one randomly firing network, where the difference in total synaptic input between the pair was minimal. That is, there was either a difference in synaptic drive (*I*_applied_) of 5 pA, or in synaptic strength (*g*_syn_) of 0.075 nS. In each network, we determined the number of cells firing in multiple bins, where the bin sizes were set to capture a full oscillation cycle of the coherent network. In this way, we determined the total number of cells firing per oscillation period, both during the firing and non-firing stages, so that this number can be justly compared between the coherent and random networks. We also determined the individual firing rates of the cells. We began our analysis at 500 ms into the simulation, as to ignore any transient effects, and analyzed bins for the remaining 1 s. Interestingly, for each pair examined (*n* = 40), we found that more cells are recruited to fire during the coherent state than during random firing, and their individual firing rates are higher, even though the difference in synaptic drive is small. For example, in the network where the standard deviation of *I*_applied_ is 12 pA, *g*_syn_ = 1.5 nS, and the mean *I*_applied_ = 595 pA (Figure 5A, bottom raster plot), 283.4 ± 8.0 cells are firing in each bin (bins are ~10 ms in width), and cells fire at an average rate of 61.4 ± 23.8 Hz. However, with only a small increase in synaptic drive (mean *I*_applied_ = 600 pA), but the same inhibitory synaptic conductance strength, 433.2 ± 8.3 cells are firing in each bin, and they are firing at an average rate of 99.4 ± 23.8 Hz. This result was consistent in networks with larger heterogeneity in *I*_applied_ as well. Thus, with a small increase in total synaptic input, many PV+ interneurons may be recruited to fire with temporal precision, greatly increasing its potential to influence network rhythms. One can test this prediction using multi-electrode recordings: given these results, one would expect to see more fast-spiking interneurons firing during HFOs than when HFOs are not present. In addition, individual PV+ cells would be expected to have higher firing rates during HFOs.

Overall, our simulation results predict that networks of CA1 fast-firing PV+ interneurons can produce high frequency population rhythms, but only when the inhibitory PV-PV synaptic conductance strengths (*g*_syn_) are relatively small (compared with approximate values derived from Bartos et al., 2002) and excitatory synaptic drive (*I*_applied_) is relatively large (compared with our experimentally estimated values). In addition, perturbation in and out of coherent states can occur abruptly given the sharp transitions obtained in our network simulations. As such, CA1 networks may be easily “gated” in and out of coherent firing regimes. Since our simulations approximate a biological context, it may be that this ease of gating in and out of coherence is an essential design property of biological networks to bring about the generation of theta/high frequency network oscillations.

## Discussion

### Summary of results and their implications

We have created a CA1 network model of PV+ interneurons that is tied to experimental work at cellular and network levels, and used it to investigate the potential of this interneuron population to realize synchronized output at high frequencies.

First, we created a model of a single PV+ interneuron based directly on experimental recordings of PV+ cells in the CA1 region of the intact hippocampal preparation *in vitro*. Due to their fast-firing properties and extensive connections with pyramidal cells (Sik et al., 1995), PV+ interneurons may play an essential role in HFOs. An experimentally constrained mathematical model of a PV+ cell allows one to investigate this role, or the role of these cells in other network states. However, to our knowledge, no other single compartment models of CA1 fast-spiking PV+ interneurons exist. Thus, we created a computationally efficient model that accurately reproduces a number of important firing properties. In order to retain computational efficiency, while representing PV+ cell spike characteristics and firing properties, we represented the PV+ interneurons with a modification of the Izhikevich-type model (Izhikevich, 2003). We used whole-cell patch clamp recordings in the presence of synaptic blockers to determine the spike characteristics and passive properties of the PV+ cells. This enabled us to constrain our model based on resting membrane potential, spike height, spike width, threshold potential, membrane capacitance, rheobase current, and amount of adaptation. In addition, a f-I profile was found, and model parameters were varied to determine the best linear fit to the slope of this f-I curve as it leveled off for higher firing frequencies. Thus, we used passive properties, spike characteristics, and firing properties of PV+ interneurons to build a mathematical model of these fast-spiking cells. As fast-firing interneurons have been proposed to play an essential role in various network oscillations (Ylinen et al., 1995; Penttonen et al., 1998; Bartos et al., 2001; Freund, 2003; Wulff et al., 2009; Colgin and Moser, 2010; Jackson et al., 2011; Belluscio et al., 2012), our model may provide a platform to investigate the influence of PV+ cells on a variety of network rhythms.

Second, we used our single cell models to construct network models, which we constrained with experimentally determined synaptic parameters, connectivity, and network size. By varying the synaptic drives and PV-PV inhibitory conductance strengths across an experimentally determined range, these network models were used to investigate the conditions under which they generated coherent high frequency firing. To create the networks, each PV+ interneuron model was connected with 60 other PV+ models (Sik et al., 1995), and synaptic properties were constrained with previously reported time constants (Bartos et al., 2002) and our own experimentally determined inhibitory reversal potential. Recordings during emergent network oscillations (Goutagny et al., 2009) provided us with information about realistic firing rates of PV+ interneurons during the “active” phase of the theta rhythm. These firing rates, used in combination with the cell's intrinsic f-I profile, provided physiological constraints on the amount of synaptic current the PV+ cells receive during their fast-firing phases in these spontaneous network oscillations. Using these experimental constraints, and exploring a physiological range of inhibitory synaptic strengths, our network model did indeed produce high frequency population rhythms (~90–200 Hz). However, in order to generate these rhythms, the synaptic drive had to be strong enough to overpower the recurrent inhibition in the network (and was therefore relatively strong compared with our experimentally determined estimates), and the PV-PV synaptic maximal conductance values were necessarily small (compared with estimates derived from Bartos et al., 2002). Thus, making the reasonable assumption that PV+ interneurons can strongly influence local field rhythms (due to their high connectivity with pyramidal cells), we predict that one should be able to induce/diminish HFOs by specifically stimulating/silencing PV+ interneurons (e.g., through optogenetic stimulation). As PV+ interneurons are selectively damaged in schizophrenia and certain drugs of abuse (Zhang and Reynolds, 2002; Lewis et al., 2005; Morris et al., 2005; Behrens et al., 2007), we predict that HFOs may be disrupted during these states. Increased excitation to PV+ interneurons may help recover this rhythm, a prediction that can be tested in these animal disease models. Thus, we suggest that PV+ interneurons may serve as a target with which one can influence the generation of HFOs—a rhythm that may play a role in memory processing (Tort et al., 2008).

In addition, we found that if we decreased the network size significantly, but maintained our connection probability, sharp transitions were lost. This occurred because in networks with a high degree of connectivity (e.g., *p* = 0.85 as in Figure 6D), the cells could fire together with small amounts of applied input (*I*_applied_). However, because *I*_applied_ was so small, only a few cells were recruited to fire, resulting in an overall low population coherence. As *I*_applied_ was increased, more and more cells were recruited to fire, and they did so together. When the connectivity was high enough, this resulted in an approximately linear increase in coherence, indicating that the cells were recruited in a somewhat linear fashion. In contrast, when the connectivity was low, the cells required relatively large levels of *I*_applied_ to synchronize. At the point of synchronization, the majority of the cells were already firing, resulting in a sharp increase in coherence. This sharp transition may play a role in the nesting of theta and HFOs, as a network of PV+ interneurons may be easily gated in and out of the coherent state. For example, if CA1 PV+ interneurons were to receive periodic excitatory drives at theta frequencies, our network model would suggest that they could easily synchronize and desynchronize at particular phases of the theta oscillation.

This finding concerning how network connectivity affects its ability to transition quickly into the coherent state may also help us understand mechanisms underlying coherent firing of PV+ interneuron networks with different connectivity properties. For example, in the CA3 region of the hippocampus, PV+ basket cells have distinct arborization compared to CA1 PV+ basket cells (Tukker et al., 2013), indicating that their network configuration may be distinct as well. Our model would suggest that networks with increased connectivity, yet similar intrinsic and synaptic properties, would not produce these sharp transitions between random and coherent population firing.

Our network models also predict that a network of PV+ interneurons exhibit sharp transitions from random to coherent firing, and that more cells fire during the coherent state. The sharp transition implies that while CA1 PV+ fast-firing cell networks have the ability to produce synchronized network rhythms at high frequencies, small perturbations to this network (from, for example, the influence of other cell types such as oriens-lacunosum-moleculare or O-LM interneurons) could easily push the system out of its coherent firing regime. Our networks showed that more cells fire, and their individual firing rates are higher, during the coherent state than during random firing, even though the change in synaptic input across this transition may be minimal. This prediction can be tested using multi-electrode recordings in the presence and absence of HFOs. Again, this would imply that HFOs would be disrupted in disease states or with particular drugs of abuse, where PV+ interneurons are selectively damaged (Zhang and Reynolds, 2002; Lewis et al., 2005; Morris et al., 2005; Behrens et al., 2007). In addition, our findings imply that PV+ interneurons increase their individual firing frequencies during HFOs. We note that the increase in individual cell firing rates and the number of cells firing are related: higher individual firing frequencies mean that each cell will be more likely to fire in a given cycle, which will directly affect the number of cells firing in each cycle.

Together these predictions imply that when firing coherently, the PV+ interneurons may have much more influence over neighboring pyramidal cells in two ways: their synaptic outputs are temporally aligned, allowing for synaptic summation, and more cells are recruited to influence postsynaptic targets. *In vivo*, it may be that CA1 inhibitory networks are easily gated in and out of coherent firing regimes with appropriate adjustments in synaptic drives, indicating a potential mechanism for the nesting of theta with HFOs. In addition, our networks suggest that PV+ interneurons form coherent assemblies, and due to their frequency and number of cells firing, may strongly influence synchrony in pyramidal cell assemblies. The timing of PV+ firing occurs at a timescale that is effective for synaptic plasticity (Magee and Johnston, 1997), and in which pyramidal cell assemblies are synchronized (Harris et al., 2003). Thus, PV+ interneurons may effectively influence information storage in neuronal circuits.

### Comparison to existing models

One of the most commonly used cellular models for fast-firing interneurons is that of Wang and Buzsáki (1996) (e.g., Bartos et al., 2001; Wulff et al., 2009; Neymotin et al., 2011), and thus it is important to consider the differences between the two models. The Wang and Buzsáki model (1996), which we will denote the “WB model,” is not representative of CA1 PV+ fast-spiking interneurons specifically, as it was developed to represent fast-firing (neocortical and hippocampal) interneurons generically, focusing on a steep f-I profile and after-hyperpolarization characteristics. As such, data from multiple preparations (e.g., rat hippocampal slices and guinea pig cortical slices; McCormick et al., 1985; Lacaille and Williams, 1990; Zhang and McBain, 1995) were considered in creating a representative model. The Hodgkin-Huxley conductance-based model framework with sodium and potassium conductances was used in creating the model.

**Figure 7:**
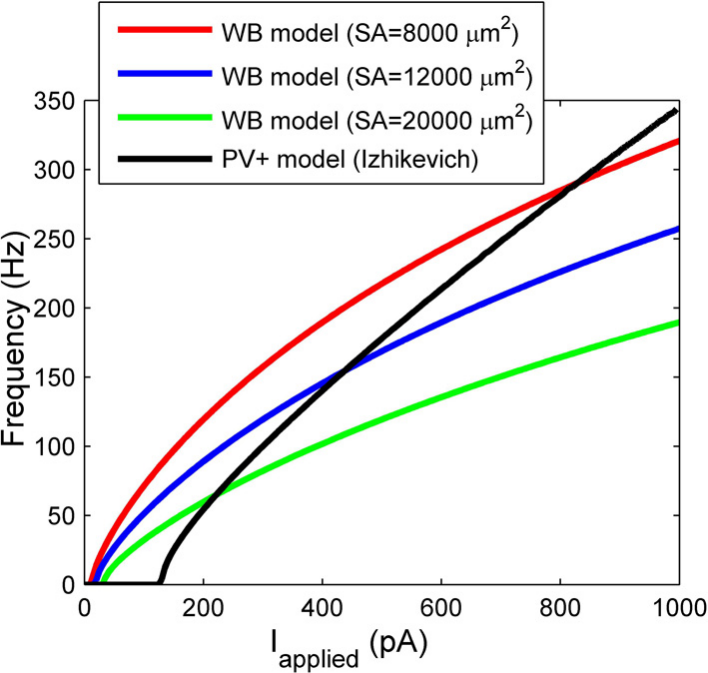
**The frequency current profiles of our model (black line) and the Wang and Buzsáki (1996) model (WB) for various surface areas (SA) (red, blue, and green lines).** Note the differences in rheobase current, and slope.

There are critical differences between our model and the much-used WB model. Our model was constrained under a specific biological setting (an intact hippocampal preparation *in vitro*), and was based on identified CA1 PV+ fast-firing interneurons. Our passive characterizations were done under different *in vitro* conditions than in studies using slices. We used an intact hippocampal preparation that better preserves neurons' axo-dendritic arborizations than conventional slice configurations, and fast aCSF perfusion rates that are critical for providing oxygenation levels required to generate network rhythmic activities (Goutagny et al., 2009). Our model uses experimental data to constrain the membrane capacitance, spike characteristics, and importantly, both the rheobase current and the slope of the f-I profile. To directly compare our f-I profile with the WB model, we consider three different somatodendritic surface areas (including one based on a reconstructed hippocampal basket cell of 12,000 μm^2^ in Bartos et al., 2001) to convert injected current values in the WB model with ours. Figure 7 shows the f-I profiles of our model (black line), vs. the WB model assuming three different surface areas. It is evident that the WB current rheobase is much smaller than ours, ~24 pA (for the 12,000 μm^2^ surface area) vs. 131 pA, which means that the WB model fires for much smaller synaptic inputs. In Ho et al. (2012), where high resolution f-I recordings were performed on fast-spiking hippocampal cells, the current rheobase was always higher than that of WB, and in agreement with our recordings (see Table 1). In addition, the slope of the WB model is not as steep as ours, and thus for the same amount of applied current, the WB model will not reach the same high frequencies.

In summary, our model differs from the WB model in its intrinsic properties including spike shape, slope of the f-I curve, and the rheobase at which it fires. Thus, our cell model would require larger excitatory inputs than the WB model in order to be recruited into a network oscillation. Given the experimental constraints under which our model was developed, it is perhaps a more accurate reflection of what is needed for network rhythms. In addition, since the slope of our f-I curve is steeper than in the WB model, our model would fire over a larger range of frequencies given a certain amount of variability in excitatory inputs.

We also consider the differences between existing network models of fast-firing interneurons (generally using WB models) (Bartos et al., 2002; Wulff et al., 2009; Neymotin et al., 2011) and our network models. Both intrinsic properties of the individual neuron models and the network properties will play a role in the network output. As mentioned above, the WB model has a less steep f-I slope relative to ours (see Figure 7). Thus, this may give rise to coherently firing output with lower gamma network frequencies. Specifically, our network models produced high frequency rhythms between ~90 and 200 Hz, compared with many other models producing rhythms within the low gamma range (Bartos et al., 2002; Wulff et al., 2009; Neymotin et al., 2011). In addition, network size and connectivity will play a role in the range of synaptic strengths required to produce coherent oscillations. Often models use smaller network sizes (Bartos et al., 2002; Wulff et al., 2009; Neymotin et al., 2011), but similar connectivity, compared to ours. As we have shown here, the sharp transitions between random and coherent firing may also be lost when connectivity is not biologically appropriate.

We note that the *I*_applied_ parameter (i.e., synaptic drive) used in previous network models producing gamma frequencies does not have any clear experimental constraint or context. For example, an *I*_applied_ value of 1 μA/cm^2^ was mostly used in Wang and Buzsáki's networks, but a value of 3 μA/cm^2^ was needed in Bartos et al.'s models when experimentally determined synaptic constraints were introduced for the inhibitory synaptic conductances and kinetics. This highlights a fundamentally different approach we used in our study—we constrained our network models with synaptic, cellular, and network level experimental data. To do this, we took advantage of the experimental context of the intact hippocampal preparation exhibiting spontaneous rhythms. Using this context, we estimated the allowable range of *I*_applied_ values (i.e., network level, synaptic drive) for our models, and used cellular models derived from the same hippocampal preparation. In addition, we used previously published synaptic conductances, kinetics, and connectivity specific to fast-firing cells in hippocampus. With this, our network models produced high frequency rhythms for particular parameter balances and with particular characteristics of sharp transitions in and out of coherence.

### Limitations

We note that, in contrast to Bartos et al. (2002), we did not include gap junction connections in our model. These connections are known to exist (Fukuda and Kosaka, 2000; Bartos et al., 2001; Deans et al., 2001; Galarreta and Hestrin, 2001) and, according to a network model by Bartos et al. (2002), gap junction conductance affects coherence selectively with only minimal effect on frequency. Thus, our model may underestimate the size of parameter range in which a network of PV+ interneurons can produce coherent rhythms. Interestingly, Hormuzdi et al. (2001) found that mice lacking connexin 36, a major neuronal connexin, resulted in disruptions of oscillations in the 30–80 Hz range, but faster (~150 Hz) oscillations remained intact. Thus, it is possible that the lack of gap junction connections in our model is not a limiting factor when considering coherence in the HFO range. In addition, we did not include spatial network architecture in our model, as was done for example in Taxidis et al. (2012). We chose not to include these details to focus on the intrinsic cellular characteristics in order to set the basis for future and more realistic network explorations.

Clearly, another limitation stems from our use of an *in vitro*, rather than an *in vivo* situation. Synaptic constraints would be expected to be different *in vivo*. However, this *in vitro* preparation does produce population rhythms spontaneously implying that synaptic constraints from it are enough for population rhythm generation. Also, assuming that spontaneously generated rhythms use mechanisms similar to those employed *in vivo*, the use of the *in vitro* preparation is advantageous. This is because the confounding issues due to dealing with more complex network circuitries as exist *in vivo* would be difficult to untangle and model to determine underlying mechanisms in the first place.

### Concluding remarks

We have combined experimental recordings, data analysis, and modeling to produce a model that is mathematically simple enough to be used in large network simulations, yet captures physiologically determined intrinsic and network properties of PV+ interneurons. Therefore, it provides a basis for understanding how hippocampal population activities involving the fast-firing PV+ cells are generated, and how they potentially contribute to other hippocampal rhythms (e.g., theta). A clear understanding of the contribution of PV+ interneurons to network rhythms is a crucial step toward determining their role in behavior. As these interneurons are thought to be involved in a variety of pathologies, including epilepsy (Maglóczky and Freund, 2005; Ogiwara et al., 2007) and dementia with lewy bodies (Bernstein et al., 2011), and are selectively damaged in schizophrenia and certain drugs of abuse (Zhang and Reynolds, 2002; Lewis et al., 2005; Morris et al., 2005; Behrens et al., 2007), this understanding of how they contribute to network rhythms becomes critically important. As such, our model provides a platform to investigate a variety of alternative questions involving the influence of PV+ interneurons.

The model may be used to address a variety of problems, but of course the usefulness of the model will depend on the question at hand. The simplicity of the model promotes computational efficiency, but questions involving the structure or detailed (e.g., ionic) properties of PV+ interneurons will require more elaborate models. Although we considered the connectivity of PV+ interneurons, we did not explore more detailed network architecture. This, along with the addition of other cell types, would be interesting extensions to our network model.

### Conflict of interest statement

The authors declare that the research was conducted in the absence of any commercial or financial relationships that could be construed as a potential conflict of interest.

## References

[B1] Aika Y. Ren J. Q. Kosaka K. Kosaka T. (1994). Quantitative analysis of GABA-like-immuno reactive and parvalbumin-containing neurons in the CA1 region of the rat hippocampus using a stereological method, the disector. Exp. Brain Res. 99, 267–276 10.1007/BF00239593 7925807

[B2] Bähner F. Weiss E. K. Birke G. Maier N. Schmitz D. Rudolph U. (2011). Cellular correlate of assembly formation in oscillating hippocampal networks *in vitro*. Proc. Natl. Acad. Sci. U.S.A. 108, E607–E616 10.1073/pnas.1103546108 21768381 PMC3167520

[B3] Bartos M. Vida I. Frotscher M. Geiger J. R. P. Jonas P. (2001). Rapid signaling at inhibitory synapses in a dentate gyrus interneuron network. J. Neurosci. 21, 2687–2698 11306622 10.1523/JNEUROSCI.21-08-02687.2001 PMC6762544

[B4] Bartos M. Vida I. Frotscher M. Meyer A. Monyer H. Geiger J. R. P. (2002). Fast synaptic inhibition promotes synchronized gamma oscillations in hippocampal interneuron networks. Proc. Natl. Acad. Sci. U.S.A. 99, 13222–13227 10.1073/pnas.192233099 12235359 PMC130614

[B5] Baude A. Bleasdale C. Dalezios Y. Somogyi P. Klausberger T. (2007). Immunoreactivity for the GABAA receptor α1 subunit, somatostatin and connexin36 distinguishes axoaxonic, basket, and bistratified interneurons of the rat hippocampus. Cereb. Cortex 17, 2094–2107 10.1093/cercor/bhl117 17122364

[B6] Behrens M. M. Ali S. S. Dao D. N. Lucero J. Shekhtman G. Quick K. L. (2007). Ketamine-induced loss of phenotype of fast-spiking interneurons is mediated by NADPH-oxidase. Science 318, 1645–1647 10.1126/science.1148045 18063801

[B7] Bekkers J. M. Delaney A. J. (2001). Modulation of excitability by alpha-dendrotoxin-sensitive potassium channels in neocortical pyramidal neurons. J. Neurosci. 21, 6553–6560 11517244 10.1523/JNEUROSCI.21-17-06553.2001 PMC6763106

[B8] Belluscio M. A. Mizuseki K. Schmidt R. Kempter R. Buzsáki G. (2012). Cross-frequency phase-phase coupling between theta and gamma oscillations in the hippocampus. J. Neurosci. 32, 423–435 10.1523/JNEUROSCI.4122-11.2012 22238079 PMC3293373

[B9] Bernstein H. G. Johnson M. Perry R. H. LeBeau F. E. N. Dobrowolny H. Bogerts B. (2011). Partial loss of parvalbumin-containing hippocampal interneurons in dementia with Lewy bodies. Neuropathology 31, 1–10 10.1111/j.1440-1789.2010.01117.x 20487308

[B10] Cobb S. R. Buhl E. H. Halasy K. Paulsen O. Somogyi P. (1995). Synchronization of neuronal activity in hippocampus by individual GABAergic interneurons. Nature 378, 75–78 10.1038/378075a0 7477292

[B11] Colgin L. L. Moser E. I. (2010). Gamma oscillations in the hippocampus. Physiology 25, 319–329 10.1152/physiol.00021.2010 20940437

[B12] Deans M. R. Gibson J. R. Sellitto C. Connors B. W. Paul D. L. (2001). Synchronous activity of inhibitory networks in neocortex requires electrical synapses containing connexin36. Neuron 31, 477–485 10.1016/S0896-6273(01)00373-7 11516403

[B13] Destexhe A. Mainen Z. F. Sejnowski T. J. (1998). “Kinetic models of synaptic transmission,” in Methods in Neuronal Modeling, 2nd Edn., eds Koch C. Segev I. (Cambridge, MA: MIT Press), 1–25

[B14] Ermentrout B. Terman D. (2010). “Synaptic channels,” in Mathematical Foundations of Neuroscience, eds Ermentrout B. Terman D. (New York, NY: Springer), 158–160 10.1007/978-0-387-87708-2_7

[B15] Freund T. F. (2003). Interneuron diversity series: rhythm and mood in perisomatic inhibition. Trends Neurosci. 26, 489–495 10.1016/S0166-2236(03)00227-3 12948660

[B16] Freund T. F. Buzsáki G. (1996). Interneurons of the hippocampus. Hippocampus 6, 347–470 10.1002/(SICI)1098-1063(1996)6:4<347::AID-HIPO1>3.0.CO;2-I 8915675

[B17] Fukuda T. Kosaka T. (2000). Gap junctions linking the dendritic network of GABAergic interneurons in the hippocampus. J. Neurosci. 20, 1519–1528 10662841 10.1523/JNEUROSCI.20-04-01519.2000 PMC6772381

[B18] Galarreta M. Hestrin S. (2001). Electrical synapses between GABA-releasing interneurons. Nat. Rev. Neurosci. 2, 425–433 10.1038/35077566 11389476

[B19] Gerstein G. L. Kiang N. Y. (1960). An approach to the quantitative analysis of electrophysiological data from single neurons. Biophys. J. 1, 15–28 10.1016/S0006-3495(60)86872-5 13704760 PMC1366309

[B20] Goodman D. F. M. Brette R. (2009). The brian simulator. Front. Neurosci. 3, 192–197 10.3389/neuro.01.026.2009 20011141 PMC2751620

[B21] Goutagny R. Jackson J. Williams S. (2009). Self-generated theta oscillations in the hippocampus. Nat. Neurosci. 12, 1491–1493 10.1038/nn.2440 19881503

[B22] Gulyás A. I. Megías M. Emri Z. Freund T. F. (1999). Total number and ratio of excitatory and inhibitory synapses converging onto single interneurons of different types in the CA1 area of the rat hippocampus. J. Neurosci. 19, 10082–10097 10559416 10.1523/JNEUROSCI.19-22-10082.1999 PMC6782984

[B23] Harris K. D. Csicsvari J. Hirase H. Dragoi G. Buzsáki G. (2003). Organization of cell assemblies in the hippocampus. Nature 424, 552–556 10.1038/nature01834 12891358

[B24] Hemond P. Epstein D. Boley A. Migliore M. Ascoli G. A. Jaffe D. B. (2008). Distinct classes of pyramidal cells exhibit mutually exclusive firing patterns in hippocampal area CA3b. Hippocampus 18, 411–424 10.1002/hipo.20404 18189311 PMC4339291

[B25] Ho E. C. Strüber M. Bartos M. Zhang L. Skinner F. K. (2012). Inhibitory networks of fast-spiking interneurons generate slow population activities due to excitatory fluctuations and network multistability. J. Neurosci. 32, 9931–9946 10.1523/JNEUROSCI.5446-11.2012 22815508 PMC6621296

[B26] Hormuzdi S. G. Pais I. LeBeau F. E. Towers S. K. Rozov A. Buhl E. H. (2001). Impaired electrical signaling disrupts gamma frequency oscillations in connexin36-deficient mice. Neuron 31, 487–495 10.1016/S0896-6273(01)00387-7 11516404

[B27] Huh C. Goutagny R. Williams S. (2010). Glutamatergic neurons of the mouse medial septum and diagonal band of Broca synaptically drive hippocampal pyramidal cells: relevance for hippocampal theta rhythm. J. Neurosci. 30, 15951–15961 10.1523/JNEUROSCI.3663-10.2010 21106833 PMC6633737

[B28] Izhikevich E. M. (2003). Simple model of spiking neurons. IEEE Trans. Neural Netw. 14, 1569–1572 10.1109/TNN.2003.820440 18244602

[B29] Jackson J. Goutagny R. Williams S. (2011). Fast and slow γ rhythms are intrinsically and independently generated in the subiculum. J. Neurosci. 31, 12104–12117 10.1523/JNEUROSCI.1370-11.2011 21865453 PMC6623234

[B30] Jinno S. Kosaka T. (2006). Cellular architecture of the mouse hippocampus: a quantitative aspect of chemically defined GABAergic neurons with stereology. Neurosci. Res. 56, 229–245 10.1016/j.neures.2006.07.007 16930755

[B31] Lacaille J. C. Williams S. (1990). Membrane properties of interneurons in stratum oriens-alveus of the CA1 region of rat hippocampus *in vitro*. Neuroscience 36, 349–359 10.1016/0306-4522(90)90431-3 2215928

[B32] Lewis D. A. Hashimoto T. Volk D. W. (2005). Cortical inhibitory neurons and schizophrenia. Nat. Rev. Neurosci. 6, 312–324 10.1038/nrn1648 15803162

[B33] Loken C. Gruner D. Groer L. Peltier R. Bunn N. Craig M. (2010). SciNet: lessons learned from building a power-efficient top-20 system and data centre. J. Phys. Conf. Ser. 256:012026 10.1088/1742-6596/256/1/012026

[B34] Magee J. C. Johnston D. (1997). A synaptically controlled, associative signal for Hebbian plasticity in hippocampal neurons. Science 275, 209–213 10.1126/science.275.5297.209 8985013

[B35] Maglóczky Z. Freund T. F. (2005). Impaired and repaired inhibitory circuits in the epileptic human hippocampus. Trends Neurosci. 28, 334–340 10.1016/j.tins.2005.04.002 15927690

[B36] McCormick D. A. Connors B. W. Lighthall J. W. Prince D. A. (1985). Comparative electrophysiology of pyramidal and sparsely spiny stellar neurons of the neocortex. J. Neurophysiol. 54, 782–806 2999347 10.1152/jn.1985.54.4.782

[B37] Miles R. Tóth K. Gulyás A. I. Hájos N. Freund T. F. (1996). Differences between somatic and dendritic inhibition in the hippocampus. Neuron 16, 815–823 10.1016/S0896-6273(00)80101-4 8607999

[B38] Morris B. J. Cochran S. M. Pratt J. A. (2005). PCP: from pharmacology to modelling schizophrenia. Curr. Opin. Pharmacol. 5, 101–106 10.1016/j.coph.2004.08.008 15661633

[B39] Neymotin S. A. Lazarewicz M. T. Sherif M. Contreras D. Finkel L. H. Lytton W. W. (2011). Ketamine disrupts theta modulation of gamma in a computer model of hippocampus. J. Neurosci. 31, 11733–11743 10.1523/JNEUROSCI.0501-11.2011 21832203 PMC3177405

[B40] Ogiwara I. Miyamoto H. Morita N. Atapour N. Mazaki E. Inoue I. (2007). Nav1.1 localizes to axons of parvalbumin-positive inhibitory interneurons: a circuit basis for epileptic seizures in mice carrying an Scn1a gene mutation. J. Neurosci. 27, 5903–5914 10.1523/JNEUROSCI.5270-06.2007 17537961 PMC6672241

[B41] Pawelzik H. Hughes D. I. Thomson A. M. (2002). Physiological and morphological diversity of immunocytochemically defined parvalbumin- and cholecystokinin-positive interneurons in CA1 of the adult rat hippocampus. J. Comp. Neurol. 443, 346–367 10.1002/cne.10118 11807843

[B42] Penttonen M. Kamondi A. Acsády L. Buzsáki G. (1998). Gamma frequency oscillation in the hippocampus of the rat: intracellular analysis *in vivo*. Eur. J. Neurosci. 10, 718–728 10.1046/j.1460-9568.1998.00096.x 9749733

[B43] Scheffer-Teixeira R. Belchior H. Caixeta F. V. Souza B. C. Ribeiro S. Tort A. B. L. (2012). Theta phase modulates multiple layer-specific oscillations in the CA1 region. Cereb. Cortex 22, 2404–2414 10.1093/cercor/bhr319 22079925

[B44] Sik A. Penttonen M. Ylinen A. Buzsáki G. (1995). Hippocampal CA1 interneurons: an *in vivo* intracellular labeling study. J. Neurosci. 15, 6651–6665 7472426 10.1523/JNEUROSCI.15-10-06651.1995 PMC6577981

[B45] Taxidis J. Coombes S. Mason R. Owen M. R. (2012). Modeling sharp wave-ripple complexes through a CA3-CA1 network model with chemical synapses. Hippocampus 22, 995–1017 10.1002/hipo.20930 21452258

[B46] Tort A. B. Kramer M. A. Thorn C. Gibson D. J. Kubota Y. Graybeil A. M. (2008). Dynamic cross-frequency couplings of local field potential oscillations in rat striatum and hippocampus during performance of a T-maze task. Proc. Natl. Acad. Sci. U.S.A. 105, 20517–20522 10.1073/pnas.0810524105 19074268 PMC2629291

[B47] Traub R. D. Whittington M. A. Colling S. B. Buzsáki G. Jefferys J. G. R. (1996). Analysis of gamma rhythms in the rat hippocampus *in vitro* and *in vivo*. J. Physiol. (Lond.) 493, 471–484 8782110 10.1113/jphysiol.1996.sp021397 PMC1158931

[B48] Tukker J. J. Lasztóczi B. Katona L. Roberts J. D. Pissadaki E. K. Dalezios Y. (2013). Distinct dendritic arborization and *in vivo* firing patterns of parvalbumin-expressing basket cells in the hippocampal area CA3. J. Neurosci. 33, 6809–6825 10.1523/JNEUROSCI.5052-12.2013 23595740 PMC4473055

[B49] Wang X. J. (2010). Neurophysiological and computational principles of cortical rhythms in cognition. Physiol. Rev. 90, 1195–1268 10.1152/physrev.00035.2008 20664082 PMC2923921

[B50] Wang X. J. Buzsáki G. (1996). Gamma oscillation by synaptic inhibition in a hippocampal interneuronal network model. J. Neurosci. 16, 6402–6413 8815919 10.1523/JNEUROSCI.16-20-06402.1996 PMC6578902

[B51] Wang X. J. Rinzel J. (1992). Alternating and synchronous rhythms in reciprocally inhibitory model neurons. Neural Comput. 4, 84–97 10.1162/neco.1992.4.1.84

[B52] Welsh J. P. Lang E. J. Suglhara I. Llinás R. (1995). Dynamic organization of motor control within the olivocerebellar system. Nature 374, 453–457 10.1038/374453a0 7700354

[B53] White J. A. Banks M. I. Pearce R. A. Kopell N. J. (2000). Networks of interneurons with fast and slow gamma-aminobutyric acid type A (GABAA) kinetics provide substrate for mixed gamma-theta rhythm. Proc. Natl. Acad. Sci. U.S.A. 97, 8128–9133 10.1073/pnas.100124097 10869419 PMC16681

[B54] White J. A. Chow C. C. Ritt J. Soto-Trevino C. Kopell N. (1998). Synchronization and oscillatory dynamics in heterogeneous, mutually inhibited neurons. J. Comput. Neurosci. 5, 5–16 10.1023/A:1008841325921 9580271

[B55] Wulff P. Ponomarenko A. A. Bartos M. Korotkova T. M. Fuchs E. C. Bähner F. (2009). Hippocampal theta rhythm and its coupling with gamma oscillations require fast inhibition onto parvalbumin-positive interneurons. Proc. Natl. Acad. Sci. U.S.A. 106, 3561–3566 10.1073/pnas.0813176106 19204281 PMC2637907

[B56] Ylinen A. Soltész I. Bragin A. Penttonen M. Sik A. Buzsáki G. (1995). Intracellular correlates of hippocampal theta rhythm in identified pyramidal cells, granule cells, and basket cells. Hippocampus 5, 78–90 10.1002/hipo.450050110 7787949

[B57] Zhang L. McBain C. J. (1995). Potassium conductances underlying action potential repolarization and after hyperpolarization in rat CA1 hippocampal interneurons. J. Physiol. (Lond.) 488, 661–672 8576856 10.1113/jphysiol.1995.sp020998 PMC1156732

[B58] Zhang Z. J. Reynolds G. P. (2002). A selective decrease in the relative density of parvalbumin-immunoreactive neurons in the hippocampus in schizophrenia. Schizophr. Res. 55, 1–10 10.1016/S0920-9964(01)00188-8 11955958

